# Liposarcoma of the Spermatic Cord With Signs and Symptoms Mimicking the Inguinal Hernia: A Rare Case Report

**DOI:** 10.1002/ccr3.70148

**Published:** 2025-01-26

**Authors:** Somar Mansour, Khedr Layka, Abdallah N. Mansour, Majd N. Mansour, Majd E. Mansour, Muhammad Sinan Muhammad, Rana Issa

**Affiliations:** ^1^ Cancer Research Center Tishreen University Hospital Latakia Syria; ^2^ Department of Pathology Tishreen University Hospital Latakia Syria; ^3^ National and Kapodistrian University of Athens Athens Greece; ^4^ Department of Genitourinary Surgery Tishreen University Hospital Latakia Syria; ^5^ Faculty of Medicine Tishreen University Latakia Syria

**Keywords:** case report, inguinal hernia, liposarcoma, spermatic cord

## Abstract

Liposarcoma of the spermatic cord (LSC) is extremely rare; < 200 cases were reported in the medical literature. Because of the rarity of these tumors and their presentation as a painless inguinal or scrotal mass, preoperative diagnosis is uncertain and they are frequently misdiagnosed as inguinal hernias.

## 1 Introduction

Liposarcomas are, rare soft tissue malignancies, composed of lipogenic cells with varying degrees of cellular atypia. These tumors originate from mesenchymal tissue and can arise from fat cells anywhere in the body [1, 2]. Liposarcoma of paratesticular tissue (spermatic cord, testicular tunics, or epididymis) is an uncommon neoplasm that comprises approximately 3%–7% of paratesticular sarcomas. This tumor was first described in 1952 and typically affects adults aged 50–60 [3, 4].

Liposarcoma of the spermatic cord (LSC) is extremely rare and < 200 cases were reported in the medical literature [1, 5]. Because of the rarity of these tumors and their presentation as a painless inguinal or scrotal mass, preoperative diagnosis is uncertain and they are frequently misdiagnosed as inguinal hernias, hydroceles, lipomas, funicular cysts, or testicular tumors [1, 4, 5].

Herein, we report a rare case of LSC presenting as an inguinal mass mimicking an inguinal hernia.

## 2 Case History/Examination

A 67‐year‐old man presented to the clinic with a complaint of a painless mass in the left inguinoscrotal region. The patient first noted the mass 2 months before. The size of the mass increased gradually during this period, leading to heaviness in the left inguinoscrotal region. There were no gastrointestinal or urinary symptoms. The patient is a smoker and drinks alcohol occasionally. His medical history was significant for uncontrolled Diabetes Mellitus II, hypertension, stable angina, and mild dyspnea.

Physical examination revealed a mass in the left inguinoscrotal region with a soft consistency. The standing position increases the size of the mass, unlike the supine position, which leads to a decrease in mass size. Trans‐illumination test was negative. Laboratory tests showed mild anemia (HGB at 12 g/dL) and elevated fasting blood glucose (160 mg/dL).

## 3 Methods (Differential Diagnosis, Investigations, and Treatment)

**FIGURE 1:**
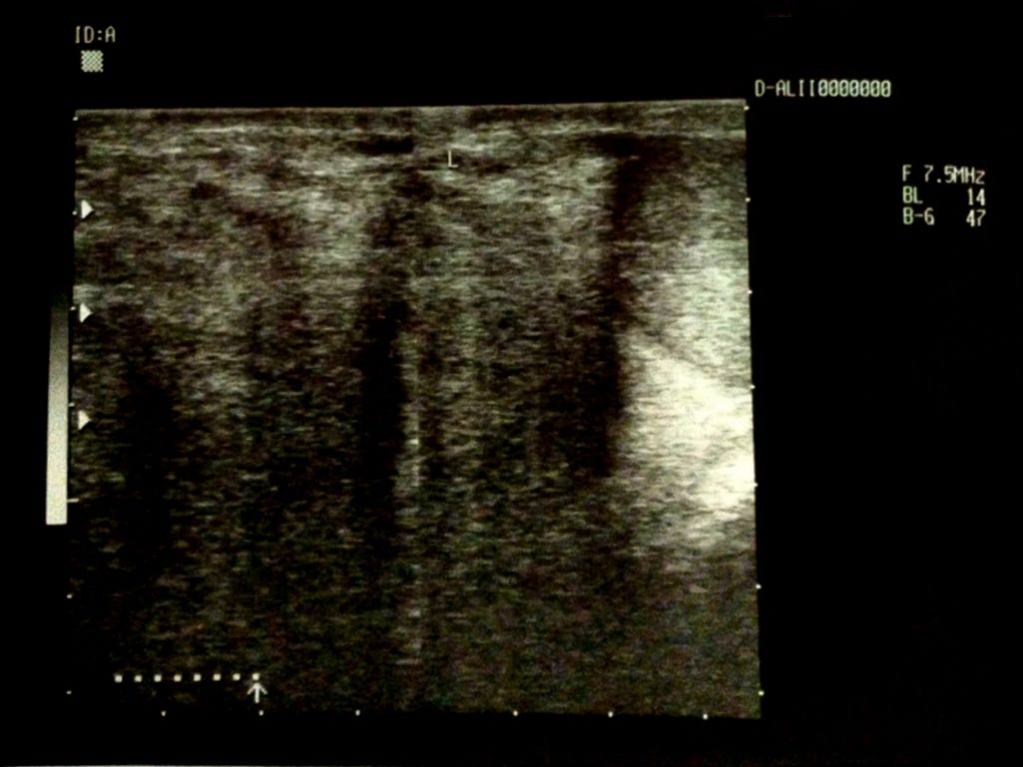
Ultrasonography shows a heterogeneous echogenic area extending from the left inguinal region to the left part of the scrotum.

Inguinoscrotal ultrasonography was conducted and showed normal testes and epididymis without local lesions. However, there was a dilation in the left spermatic veins measuring 4 mm and regurgitation of blood flow correlating with grade III spermatic varicocele. Additionally, an ultrasound of the inguinal mass showed a heterogeneous echogenic area extending from the left inguinal region to the left part of the scrotum (Figure 1). The hypoechoic portion of the mass is consistent with the presence of an intestinal loop. The diagnosis of inguinal hernia was suspected based on the clinical and ultrasonography findings.

After discussing the treatment options with the patient, surgical management was decided and performed. An incision parallel to the left inguinal ligament was made under lumbar anesthesia and upon reaching the spermatic cord, the surgeon found an ill‐defined mass rather than an intestinal loop. Therefore, a genitourinary surgeon was consulted to excise the mass by radical orchiectomy. The left testis and spermatic cord with the surrounding mass were isolated and resected. After that, a zigzag drainage was put in the scrotum and the incision was closed on layers successfully.

**FIGURE 2:**
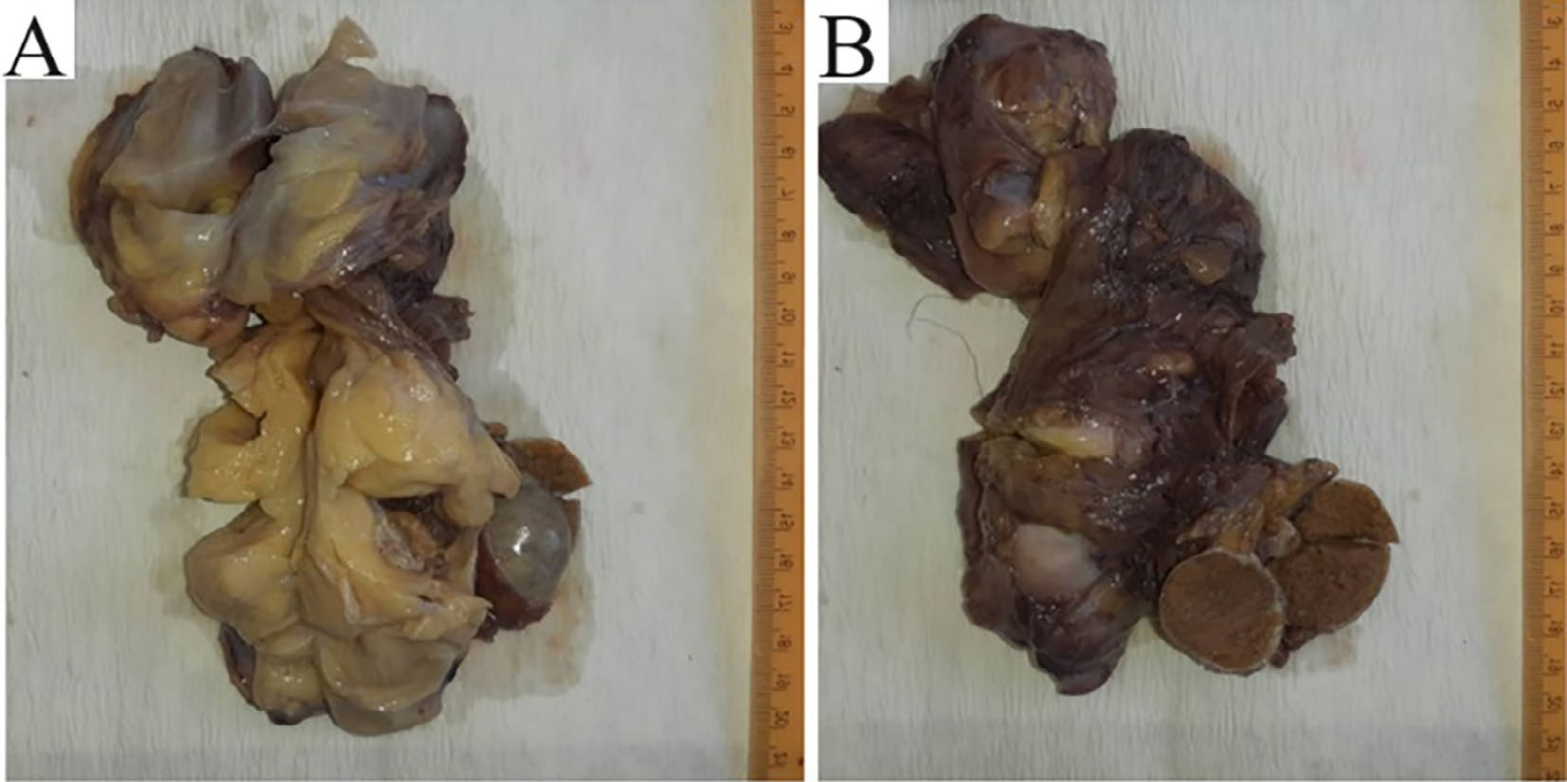
Gross appearance of the resected mass; (A) multilobulated formations of yellow lipoid tissue with bright myxoid areas and (B) left spermatic cord mass with normal testis.

Grossly, the resected specimen measured (15 × 12 × 5) cm and consisted of a normal testis with a spermatic cord surrounded by multilobulated yellow lipoid tissue separated by variably wide gray‐white connective tissue and showed bright myxoid areas on cut sections (Figure 2). However, the status of the upper resection lines of the lipoid tissue was unrecognizable and could not be evaluated.

**FIGURE 3:**
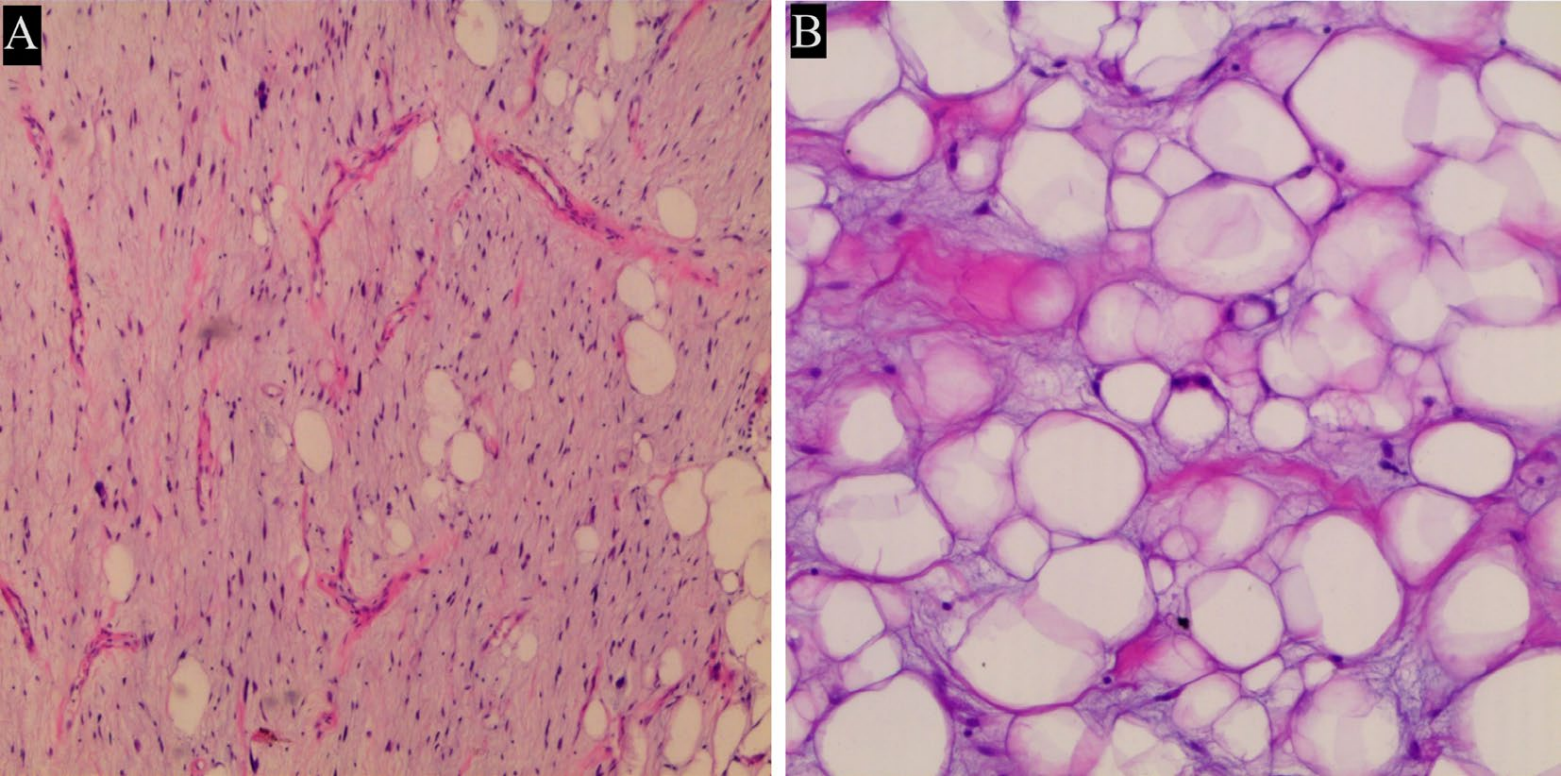
Histopathologic examination of the specimen. (A) H&E section on 40× magnification shows mildly atypical spindle cells dispersed in a myxoid stroma with arborizing thin‐walled vessels; (B) H&E section on 100× magnification shows signet ring lipoblasts.

H&E stained sections revealed the proliferation of variably sized lipocytes separated by fibrous collagenous septa containing mono‐ and multinuclear atypical cells. The grossly seen myxoid areas composed of mildly atypical spindle cells and signet ring lipoblasts dispersed in a myxoid stroma with delicate arborizing vasculature and showed resemblance to the chicken wire pattern seen in myxoid liposarcoma (Figure 3). Finally, based on the constellations of gross and microscopic findings, the diagnosis of well‐differentiated Liposarcoma with prominent myxoid stroma of the spermatic cord was reported.

## 4 Conclusions and Results (Outcome and Follow‐Up)

The patient has been redirected to the oncology department to irradiate the tumor site because the status of the surgical margins could not be assessed. However, the patient refused to receive chemotherapy and was released. No recurrence was detected during his follow‐up visit.

## 5 Discussion

The inguinal region is anatomically complex, containing multiple structures, which makes swellings in this area frequently mistaken for inguinal hernias. When differentiating inguinal masses, it is essential to categorize them based on the patient's age group, as certain conditions are more common in specific stages of life. The five primary categories include congenital anomalies (more frequent in newborns and infants), acquired hernias (typically in adults and the elderly), vascular disorders (seen more in middle‐aged individuals), infections or inflammatory conditions (common in children and young adults), and tumors, which can occur at any age but are of particular concern in older adults due to the increased risk of malignancy [6]. Given the focus on a younger population, attention should also be given to benign masses such as lipomas.

Liposarcomas are a type of soft tissue sarcomas that occurs predominantly in the pelvic and retroperitoneal regions, with the paratesticular area being a less common site, accounting for approximately 12% of all cases. Specifically, paratesticular liposarcoma (PLS) constitutes about 7% of scrotal tumors, which is an uncommon occurrence in the context of soft tissue tumors in the testicular and paratesticular regions [7].

Liposarcomas typically affect adults, with the highest incidence observed between the ages of 40 and 60, and they are slightly more prevalent in men [8]. Clinically, liposarcoma of the testis often presents as a painless mass, although in some instances, it may manifest as a rapidly enlarging scrotal mass [9].

For the diagnosis of PLS, CT scans with contrast are preferred. Well‐differentiated liposarcomas (WDLPS) typically exhibit clear margins, a striated or lobular contour, and predominantly fat attenuation without calcification. The presence of internal nodular septations with mild to moderate enhancement is a key feature that helps to differentiate WDLPS from benign lipomas. Additionally, liposarcomas with myxoid (mucinous) regions may show different imaging characteristics on CT, such as lower attenuation, which can help distinguish them from other types of soft tissue tumors [9]. Despite the importance of CT imaging, in this particular case, the patient did not undergo axial tomography. However, an ultrasound was performed, revealing a mixed‐echo mass with fatty components. In our case, the detection of a heterogeneous echogenic area, initially suspected to be an intestinal loop, highlights the potential for misdiagnosis when relying solely on sonography. Initially, the surgery was performed under the assumption of a hernia, but the intraoperative findings led to the involvement of a urological surgeon, underscoring the complexity and rarity of this diagnosis.

The rarity of liposarcoma in the spermatic cord is significant, as this type of tumor more commonly occurs in the retroperitoneum and extremities [10]. Recent genetic studies have identified distinct molecular profiles in liposarcomas: well‐differentiated liposarcomas exhibit high levels of TIMP‐4 and low YAP/TAZ expression, whereas dedifferentiated liposarcomas show elevated TIMP‐1 and activated YAP/TAZ levels [8].

Surgery remains the cornerstone of treatment for spermatic cord liposarcoma. While adjuvant therapies like radiation and chemotherapy can be considered, their effectiveness is generally limited, and no standardized treatment protocol exists. A high orchiectomy, which includes the removal of surrounding normal tissue, is essential for achieving clear margins. Simple tumor resection often proves inadequate due to the increased risk of local recurrence and distant metastases, which significantly influence prognosis [8].

The role of adjuvant radiotherapy or chemotherapy in the management of PLS continues to be debated. Studies suggest that adjuvant radiation does not significantly impact recurrence‐free survival, and chemotherapy's efficacy is limited. For dedifferentiated liposarcoma, first‐line chemotherapy response rates are around 25%, with anthracycline‐based treatments showing only a 12% effectiveness. Although evidence is scarce, systemic chemotherapy might offer survival benefits in advanced cases [7].

In this case, after the initial diagnosis, the patient underwent further imaging, including MRI and CT scans, to assess the tumor's remnants and any potential spread, which revealed no additional findings. Post‐operatively, the patient was closely monitored, and follow‐up evaluations showed no signs of recurrence. Despite the patient's refusal to undergo chemotherapy, routine follow‐up over 12 months revealed no recurrence. This outcome raises concerns about the long‐term prognosis, given the malignant nature of liposarcoma and its potential for local recurrence, metastasis, and dedifferentiation. Ensuring adherence to recommended oncological treatments is crucial for optimizing outcomes [11].

## 6 Conclusion

Liposarcomas are rare soft tissue malignancies with varying degrees of cellular atypia. Liposarcoma of the spermatic cord (LSC) is extremely rare and can be easily misdiagnosed for inguinal hernias, hydroceles, lipomas, funicular cysts, or testicular tumors. We report a rare case of LSC presented as an inguinal mass mimicking an inguinal hernia and discuss its diagnostic challenges.

## Author Contributions

**Somar Mansour:** conceptualization, writing – original draft. **Khedr Layka:** resources, writing – original draft. **Abdallah N. Mansour:** writing – original draft, writing – review and editing. **Majd N. Mansour:** writing – original draft, writing – review and editing. **Majd E. Mansour:** writing – original draft. **Muhammad Sinan Muhammad:** writing – review and editing. **Rana Issa:** supervision, writing – review and editing.

## Consent

Written informed consent was obtained from the patient to publish this case report.

## Conflicts of Interest

The authors declare no conflicts of interest.

## Data Availability

Data and material are available on reasonable request from the corresponding author.
